# Low Concentrations of Flavonoid - Rich Fraction of Shallot Extract Induce Delayed - Type Hypersensitivity and TH1 Cytokine IFNγ Expression in BALB/c Mice

**Published:** 2014

**Authors:** Leila Farhadi, Hamid-Reza Mohammadi-Motlagh, Parivash Seyfi, Ali Mostafaie

**Affiliations:** 1 *Department of Immunology, School of Medicine, Kermanshah University of Medical Sciences, Kermanshah, Iran.*; 2 *Medical Biology Research Center, Kermanshah University of Medical Sciences, Kermanshah, Iran.*

**Keywords:** *Allium ascalonicum*, immunomodulation, delayed-type hypersensitivity, interferon-γ, hydroalcoh-olic extract

## Abstract

Flavonoids are potentially immunomodulatory factors and it may be inferred that these phytochemicals contribute to immunomodulatory properties of the* Allium* family. In the present study, we investigated the potential mechanism underlying the immunomodulatory effect of shallot and its ethyl acetate (EA) fraction as flavonoid-rich sources. *Ex vivo*, effects of a hydroalcoholic extract of shallot, its fractions and quercetin on lymphocyte viability were evaluated. The proliferative effects of the fractions were examined using naive mouse lymphocytes to determine the fraction with highest impact/ activity. In addition, in a mouse model, both delayed- type hypersensitivity (DTH) responses and production of a key cytokine (interferon [IFN]-ᵧ) were evaluated. Both the shallot extract and its fractions inhibited lymphocytes cell growth and survival in a concentration- dependent manner. The findings also showed that the extract and especially the ethyl acetate (EA) fraction could induce lymphocyte proliferation. The evaluation of the extract and its EA fraction on DTH responses indicated that both caused a significant increase in DTH response. Furthermore, they triggered significant increases in IFNγ and decreases in interleukin (IL)-4 production by splenic mononuclear cells. Because of the significant immunomodulatory activity displayed in these studies, it is plausible that shallot could have a potential use as an immunomodulatory agent in clinical settings.

## Introduction

CD4 T-cells play a central role in the immune system function through their capacity to help B-cells, to enhance and maintain responses of CD8 T-cells, to regulate macrophage function and orchestrate immune responses against a wide variety of pathogenic microorganisms (1). During T-cell receptor (TCR) activation in a particular cytokine milieu, naive CD4 T-cells polarize into lineages of T-helper (T_H_) cells, including T_H_1, T_H_2, T_H_17, and iT_reg_, as defined by their individual cytokine production patterns and function (2-4).

The molecular basis of T_H_1 cell differentiation involves the interplay of signals among T-cell receptors (TCR), the cytokines interferon (IFN)- ᵧ and interleukin (IL)-12, and the transcription factors T-bet, STAT1 and STAT4 (5). On the other hand, the basis of T_H_2 cell differentiation involves interplay of signals among the TCR, IL-4, and transcription factors GATA-3 and STAT6 (6). Recent studies have shown that T_H_1 cell-derived cytokines like IFNᵧ and IL-12 promote cellular immunity, while T_H_2 cell-derived agents like IL-4 and IL-5 induce humoral immunity (7-8). In this regard, T_H_1 cells are the major inducer of delayed-type hypersensitivity (DTH) responses since they secrete potent stimulators of macrophage functions.

Natural compounds present in/derived from medicinal plants display diverse pharmacologic activities and generally have advantages over many synthetic drugs, including having smoother action and a better tolerance (9). For example, quercetin, a flavonoid found in *Liliaceae* family and many other food plants, has potent effects on lymphocyte proliferation and can regulate cell-mediated immunity. In addition, isoliquitrin, ginsenoside  and torilin are flavonoids that have potent effects on endothelial cell proliferation or tube formation (10-13).

The shallot (*Allium ascalonicum*) is a major component of many Asian diets and is widely believed to be beneficial for health. Like garlic and other members of this family, shallot contains biologically-active components including organo-sulfur compounds, polyphenols and selenium (14). To date, there are few clinical reports about the pharmacologic properties of shallot. These include: *in vitro* analyses of the anti-oxidant / anti-bacterial activities of shallot and *in vivo* analyses of the hypoglycemic effect of aqueous extracts of shallot (and garlic) in rats with fructose induced insulin resistance (15-17 ). Shallots contain considerable amounts of flavonoids that can increase the proliferative activity of lymphocytes *in vitro* (18). Quercetin is found in high concentrations in shallot  and can exert significant immuno-modulatory effects, in part, by modulating the  production of T_H_1 and T_H_2 cell-derived cytokines (19).

Previously, we indicated that a flavonoid-rich fraction of a hydroalcoholic extract of shallot could abolish angiogenesis *in vitro* and *in vivo* (20). Furthermore, we reported the results of studies that delineated the *in vitro* anti-cancer and *in vivo* anti-inflammatory activities of shallot extract (21). Building upon those earlier findings, the studies here sought to examine the effects of a hydroalcoholic extract of shallot and its flavonoid-rich fraction, on  induction of delayed type-hypersensitivity (DTH) responses and production /  release of a key T_H_1 cytokine (i.e., IFNᵧ) in BALB/c mice.

## Materials and methods

**Mice**

BALB/c mice (male, 6-8 weeks old) were obtained from Pasteur Institute of Iran (Tehran, Iran). All mice were housed in cages (4-5/cages)  located in our center (MBRC) facilities maintained at 28°C with a 50% relative humidity and a 12- hr light / dark cycle. All mice had *ad libitum* access to standard rodent chow and filtered water throughout the study. The mice were housed for one week  to acclimate prior to any experiments. The Animal Care and Use Protocol Committee of Kermanshah University of Medical Sciences (Kermanshah, Iran) approved all experiments and protocols performed in these studies.

**Preparation** **of shallot bulb hydroalcoholic extract and other organic fractions**

Shallot bulbs were purchased from a local vegetable market at Kermanshah and verified at the Agricultural College of Razi University . The preparation of the hydroalcoholic extract of shallot and other fractions was performed using successive fractionation (22-23). In brief, the bulbs were homogenized and extracted with 50% (v/v) ethanol by stirring at 4°C for 24 hr. The extract was filtered through Whatman paper No.1 and the filtrate was centrifuged (12,000×g, 20 min, 4°C) to remove any debris. The cleared supernatant was then allowed to evaporate to dryness under reduced pressure. To permit solvent fractionation, the powder was re-suspended in distilled water and then partitioned successively with *n*-hexane (Hex), ethyl acetate (EA), and n-butanol (BuOH), until only a residual aqueous fraction (Aq) remained. Each isolated fraction was evaporated under reduced pressure to yield what were termed the Hex, EA, BuOH and Aq fractions.

For calculating quantitative yields, both parent  (hydroalcoholic) extract and each sub-fraction were dried  and then weighed. These values were used to determine the  relative contribution of each sub-fraction to the total parent extract mass and for the preparation of individual materials for use in *in vivo * and* in vitro* studies.

**Toxicity** **of the hydroalcoholic** **extract** **of shallot in the mouse**

To evaluate any potential *in situ* toxic effects of the hydroalcoholic extract of shallot, the mice were weighed and then doses ranging from 10-2000 mg hydroalcoholic extract/kg were injected intraperi-toneally (IP) in a 100-200 µl volume for five consecutive days. These particular doses were selected based on the findings resulted from the previous experiments (20, 24-25). The mice were monitored for any changes in outward appearance and/or behaviors (loss of preening, sluggishness, diarrhea, etc.) over the course of exposures . At 72 hr after the final injection, the mice were euthanized by cervical dislocation and then their spleens and other major organs (e.g, liver and kidney) were removed, fixed in 10% (v/v) paraformaldehyde, dehydrated and then embedded in paraffin. For histological examination, 4-μm sections of the fixed tissues were cut using a Leica model 2165 rotary microtome (Leica, Nussloch, Germany) and placed on slides. The sections were then de-paraffinized, sequentially stained with hematoxylin-eosin Y (Richard-Allan Scien-tific, Kalamazoo, MI) and  then evaluated microscopically.

**Cytotoxicity and proliferation assays**

The cytotoxic effects of the shallot extract (as well as its fractions) on the mouse spleen mono-nuclear cells (MNC) were evaluated. Briefly, naïve mice were euthanized (by cervical dislocate-ion) and their spleens were  removed and mechanically disrupted in phosphate-buffered saline (PBS, pH 7.4) under sterile conditions. The resulting suspension was passed through a 100-µm stainless steel mesh and red blood cells (RBC) present were removed by incubation for 15 min on ice in lysis buffer (150 mM NH_4_Cl, 1 mM KHCO_3_  and 0.1 mM Na_2_EDTA) followed by centrifugation (3000×g, 5 min, 4°C). The cells were washed twice with PBS and re- suspended in 1 ml RPMI 1640 containing 10% fetal bovine serum (FBS), 5 μg Concanavalin A (ConA)/ml, 100 U penicillin/ml and 100 µg streptomycin/ml (all reagents were  purchased from Sigma [St. Louis, MO]). After counting the cells, aliquots containing 10^5 ^cells were placed into each well of a 24-well plate and the plate was held at 37°C in a 5% CO_2_ incubator for 24 hr. Thereafter, different doses of the shallot extract or its fractions were added to designated wells; after further 48 hr incubation, the cells were washed with PBS, harvested  and evaluated for viability via trypan blue exclusion.

Cell proliferation was estimated using an MTT assay according to the method of van de Loosdrecht et al. in 1994 (26). In brief, the isolated cells were seeded (5×10^4^ cells/well ) in 96-well microplates  and different doses of shallot extract or its fractions were then added (in 25 µl volumes) to dedicated wells. After 24 hr incubation at 37°C in 5% CO_2_, the cells were stimulated by addition of Con A (final concentration 5 µg/ml). After 48 hr of incubation, 50 μl MTT (3-(4,5-dimethylthiazol-2-Yl)-2,5-diphenyltetrazo-lium bromide; Sigma) was added to each well (to yield final 5 µg/ml concentration). After further 4 hr incubation, the medium in each well was removed and 250 μl DMSO (dimethyl sulfoxide, 99%) was added to solubilize the formazan crystals that formed within live/ proliferating cells. The absorbance of the formazan dye present in each well was measured at 570 nm using a Stat Fax 2100 plate reader (Aware-ness Technology, Inc., Palm City, FL).

**DTH** **response**

DTH responses were evaluated by priming naïve mice with 10^8 ^sheep red blood cells (SRBC; obtained from Aburaihan Pharmaceutical Company, Tehran) in a 0.1 ml volume injected subcutaneously (SC) into the base of their tail on Day 0 (sensitization stage). On Day 1, the sensitized animals were randomized into groups (n=5/group) and treated with 100, 1000 or 2000 mg hydroalcoholic extract of shallot/kg/d, 0.1, 0.5 or 2.5 mg EA fraction/kg/d, or 20 or 40 mg quercetin/kg/d for five consecutive days (each time in a 250 µl IP injection). The controls received IP injections of PBS buffer only. Within 1 hr after the final treatment, each mouse was challenged with 10^8 ^SRBC injected SC into the left hind footpad (elicitation). Increases in the thickness of the treated animals’ footpad (compared to untreated animals’ footpad) were monitored after 24 and 48 hr using vernier calipers (27).

**Cytokine assay**

To evaluate any effects of the shallot extract or the EA fraction on cytokine production by splenic MNC after host treatment with the test materials, the MNC in spleens of hosts treated with the test materials as mentioned  above (for five days; n = 5/group) were isolated using protocols outlined above for naïve mice samples. After determining the viability of the cell isolates, 5×10^6^ live cells/ml were aliquoted (in 100 µl volumes) into wells of a 96-well microplate  and then stimulated by addition of 100 µl medium containing 10 µg ConA/ml (for 5 µg ConA/ml final concentration). The plate was then incubated at 37°C in a 5% CO_2_ incubator for 8 hr. At that point, supernatants in each well were harvested for the determination of IFNᵧ and IL-4 levels. All samples were analyzed using commercial ELISA kits (R&D Systems, Minnea-polis, MN) according to manufacturer's protocols. The sensitivity of both the IFNᵧ and IL-4 kits was 2 pg/ml.

**Statistical analysis**

All data were presented as mean ± SE. Statistical analyses were performed for each endpoint using a Student’s *t*-test and a one-way analysis of variance (ANOVA). P-values  < 0.05 were considered statistically significant.

## Results

**Fractions of shallot**

The bulbs of shallots (≈ 200 g) were extracted with 50% (v/v) ethanol and concentrated to dryness under reduced pressure to produce a hydroalcoholic extract (≈ 42 g). The extract was then successively fractionated into n-hexane (0.35% of parent extract, by mass), ethyl acetate (0.75%), n-butanol (7.9%), and aqueous (91%) fractions.

**Toxicity** **effect** **of shallot** **extract** **on mouse**

The results indicated that a total of five repeated IP injections of hydroalcoholic extract of shallot, at concentrations of up to 2000 mg/kg, had no toxic effect on mice  during the course of the exposures and even up to a period of 72  hr after the final injection. Microscopic analyses indicated that no  significant pathological changes in spleen and other key organs were induced under these conditions at any of the doses tested (data not shown).

**Effect of shallot and its fractions on viability and growth of mouse splenic MNC**

**Fig 1:**
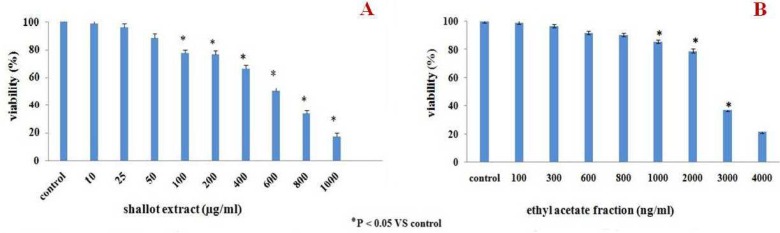
*In vitro* cytotoxicity of** (A)** hydroalcoholic extract of shallot or **(B)** EA fraction in mouse splenic mononuclear cells (MNC). The extent of cell viability after 48- hr  treatment was evaluated by trypan blue exclusion. The values shown are the means (± SEM) of three independent experiments. *P <  0.05

MNC viability as a result of *in vitro* exposure to the test extract/fractions was evaluated using trypan blue exclusion. The results indicate that the mouse MNC retained viabilities of > 80% at doses up to 200 µg shallot extract/ml (Figure 1A) and viabilities > 80% even up to 2000 ng EA fraction/ml (Figure 1B). The lethal concentrations (LC_50_) of the parent hydroalcoholic extract and the EA fraction were estimated to be  600 and 2.5 µg/ ml respectively  (Table 1). The LC_50_ values for the Hex, But and Aq fractions and quercetin were also calculated. Only the Aq imparted a weaker cytotoxic effect than the parent extract; the other LC_50_ values were on the order of quercetin > But > Hex, with Hex being only slightly less toxic to cells than the EA fraction.

**Fig 2:**
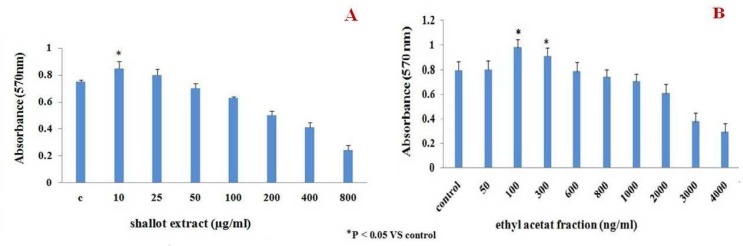
*In vitro* effect of **(A)** hydroalcoholic extract of shallot or **(B)** EA fraction on proliferation of naïve mouse splenic MNC. Cell proliferation was estimated using an MTT assay. The data shown are the means (± SEM) of three independent experiments. *P <  0.05

The studies here also examined the effects of the hydroalcoholic extract of shallot and its fractions (and also quercetin, as positive control) on proliferation of lymphocytes  within the MNC populations. The results indicated that the shallot extract at 10 and 25 µg/ ml led to increases in ConA-induced lymphocyte proliferation (Figure 2A). The EA fraction, which had the highest activity among all the shallot sub-fractions, could significantly induce the proliferation at doses of 100 and 300 ng/ ml (Figure 2B). The other fractions, as well as the quercetin, had proliferative effects that mirrored the trends noted in the cytotoxicity outcomes (data not shown).

**Effect of shallot extract and its EA fraction on DTH response**s

**Fig 3:**
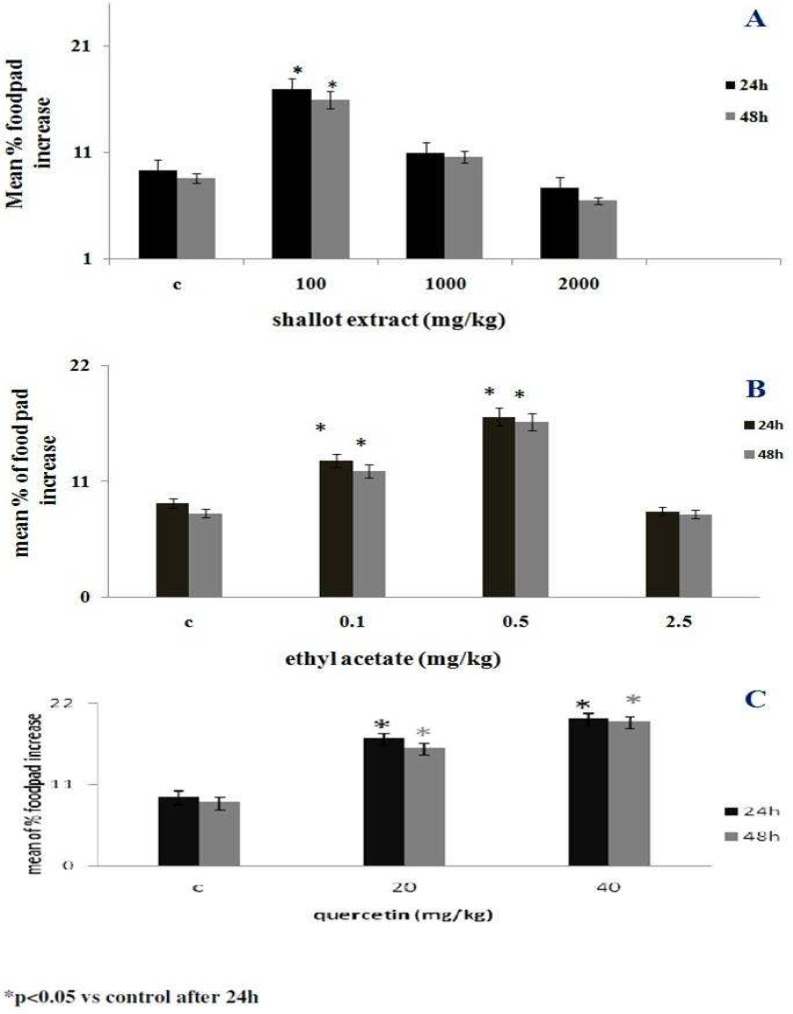
Effects of (A) hydroalcoholic extract of shallot, (B) EA fraction or (C) quercetin (as standard flavonoid) on DTH responses. The mice were sensitized to SRBC and then subjected to five daily host treatments with the test agents. Within 48 hr after the final treatment, each mouse was challenged in one footpad with SRBC (the other received saline). Pad thickness was measured 24 and 48 hr later and compared to that in the host- matched saline- injected footpad. The data shown are the means (± SEM) of three independent experiments. N = 5/group. *P <  0.05

To evaluate the effects of the parent shallot extract and the EA fraction on DTH responses, the thickness of SRBC (antigen)-treated host’s footpads were compared to those of the untreated host’s footpads after 24 and 48 hr. The results indicated that exposure to the hydroalcoholic extract of shallot at 100 mg/kg caused a significant increase in DTH response even within 24 hr (Figure 3A). The results also indicated that the EA fraction at 0.1 and 0.5 mg/kg could induce significant DTH responses (Figure 3B). Quercetin (as a standard flavonoid), displayed a significant effect at doses of 20 and 40 mg/kg (Figure 3C).

**Table 1:** *In vitro* effects of the hydroalcoholic extract of shallot, its sub- fractions and quercetin on mouse MNC survival. The LC_50_ for each fraction is reported

**Sample**	**IC**_50_ ** (µg/ml)**
Shallot extract (hydroalcoholic)	600
Hexan fraction	16
Ethyl acetate fraction	2.5
Butanol fraction	40
Aqueous fraction	700
Quercetin	300

**Fig 4:**
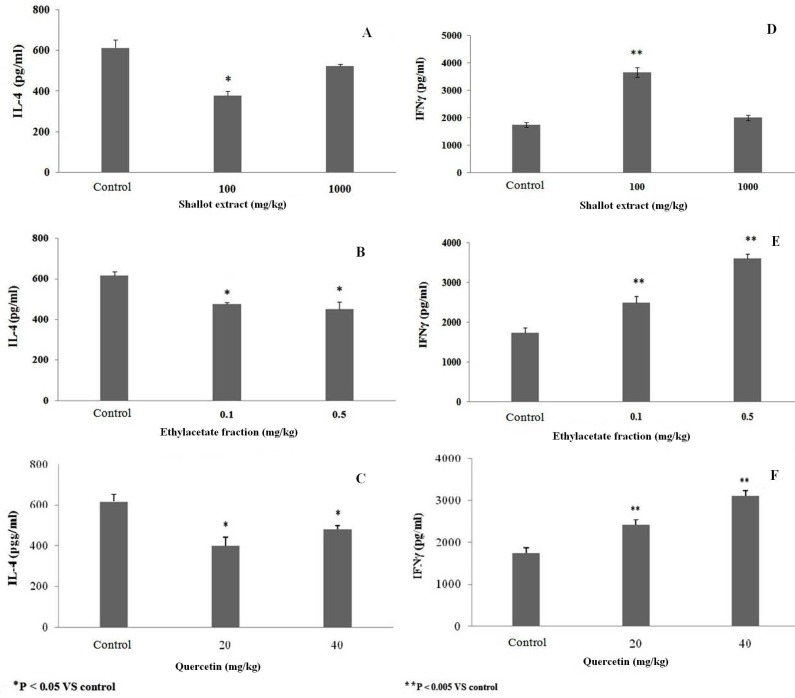
Effect of (A, D) hydroalcoholic extract of shallot, (B, E) EA fraction or (C, F) quercetin (as standard flavonoid) on  *ex vivo* IFNγ and IL- 4 formation by splenic MNC. MNC were isolated from mice after following the last of five daily host treatments with the test agents. The data shown are the means (± SEM) of three independent experiments. N = 5/ group. *P <  0.05 vs. control

**Effects of shallot extract or its EA fraction on IL- 4 and IFN**ᵧ** production by splenic MNC**

With respect to TH2-cell derived IL-4, treatments with the shallot extract at 100 mg/kg and the EA fraction at 0.1 or 0.5 mg/kg significantly decreased the amounts of IL-4 produced by MNC isolated from treated mice compared to that by MNC of control mice (Figure 4 A, B). Quercetin significantly inhibited this production when used at doses of 20 and 40 mg/kg (Figure 4 C).

Levels of IFNᵧ in the culture supernatants of splenic MNC isolated from mice that received repeated injections of different concentrations of shallot extract or its (EA) fraction were evaluated. The ELISA data shown in Figure 4 (D, E) illustrates how the parent extract at 100 mg/kg and the EA fraction at 0.1 and 0.5 mg/kg each triggered significantly increased production of IFNγ by cells from the treated mice as compared to that by cells from control counterpart hosts. Quercetin (standard flavonoid) also significantly induced IFNγ formation, but when given at a dose of 20 or 40 mg/kg (Figure 4F).

## Discussion

The results of several population- based studies have indicated that intake of *Allium* vegetables is inversely associated with the risk of cardiovascular, diabetes and infectious diseases as well as certain types of cancer (28). Additionally, several studies triggered intense research in the past two decades that aimed not only to identify the putative phytochemicals responsible for the aforementioned health benefits of *Allium* vegetables but also to elucidate the mechanisms of action (29).

Shallot, as a major component of many Asian diets, is widely believed to be beneficial to health. Furthermore, it contains flavones and polyphenolic derivatives with anti- cancer and anti- angiogenic properties (30-31). In previous studies, we showed that a hydroalcoholic extract of shallot and its fractions (especially an ethyl acetate (EA) fraction) exhibited significant anti-angiogenic effects *in vitro*, *ex vivo* and also in *in vivo* models (20, 24-25). Additionally, we demonstrated that heat and low pH has had no effect on anti- angiogenic activity of this extract or the EA fraction (20, 24). Furthermore, we observed that the aqueous extract of shallot imparted a considerable anti- inflammatory activity in a mouse model and anti-growth effects against cancer cells* in vitro*, i.e, Jurkat, K562 and Wehi-164 (21).

In contrast to the inflammation, DTH is a reaction mediated by T-cells and is associated with activation of macrophages and effector T-cells. The T_H_1 cell is the principal "inducer" of a DTH response, in part, through secretion of IFNᵧ which is a potent stimulator of macrophages. The process of activation of macrophages and natural killer (NK) cells as well as cytotoxic T-cells stimulation by cytokines released from T_H_1 cells are an important protective mechanism against intrace-llular pathogens (32).

In the present study, it was seen that a hydroalcoholic extract of shallot and its EA fraction each could cause a significant increase in DTH response in mice. In addition, the results showed that each agent could induce significant increases in IFNᵧ production, and decreases in IL-4 production, by splenic MNC from hosts treated with the test materials. Our laboratories had shown previously that the EA fraction which was rich in flavonoid compounds, imparted significant inhibitory effects on angiogenesis (20). In that study, it was seen that a hydroalcoholic extract of shallot had a large flavonoid content. Interestingly, the EA fraction had the highest amount of flavonoid compounds (followed by the Hex, BuOH and Aq fractions). As such, the effects reported here and in our earlier works would be in keeping with what is known about agents rich in flavonoids/related agents that also act as potential immunomodulants.

Leighton et al. in 1992 (33) showed that shallot contain the highest levels of total flavonols among all onion varieties. However, the bulbs of shallot also have high concentrations of quercetin, isorhamnetin and their glycosides (34). It has been reported that flavonoids increase proliferation of lymphocytes isolated from mouse spleen *in vitro* (18). Yu et al. in 2010 (35) showed that quercetin inhibited murine leukemia, in part, by modulating the host immune response. It was subsequently shown by those investigators that these outcomes resulted in great part from a stimulation of macrophage phagocytosis and an overall promotion of natural killer (NK) cell activity. It was shown here that the EA fraction imparted significant effects on DTH induction and on IFNᵧ production by MNC. Based on the role of macrophages in DTH responses and IFNᵧ on NK cell activity, the two outcomes reported in our study can be assumed linkable to the Yu et al. noted effects on macrophage and NK cells.

Interestingly, a significant difference was seen between the effective concentrations of the EA fraction when compared to that determined for pure quercetin (0.1-0.5 vs. 20-40 µg/ml). These remarkable differences could potentially be attributable to suspected additive effects of flavonoid compounds (such as quercetin and isorhamnetin in unconjugated or conjugated forms) that are naturally present in the shallot and still within its EA fraction. By this, the various members of this class of compounds that are in the hydroalcoholic extract of shallot/EA fraction might act synergistically (or additively) at low (i.e, ng) doses to impart effects that would not be achieved until µg to mg doses of either pure single agent (like quercetin) alone. Whereas to be determined the molecular weight and concentrations of flavonoids compounds, one can conclude with high certainty that the ethyl acetate fraction rich in flavonoids of shallot is effective even at “micromolar” concentrations examined in this study. Our latest ongoing studies are also exploring to purify and identify the efficient compound of shallot. Further studies are clearly ongoing to clarify the exactly responsible component(s) and their mechanisms of effect.

## References

[1] Zhu J, Yamane H, Paul WE ( 2010). Differentiation of effector CD4 T cell populations (*). Annu Rev Immunol.

[2] Mosmann TR, Coffman RL ( 1989). TH1 and TH2 cells: different patterns of lymphokine secretion lead to different functional properties. Annu Rev Immunol.

[3] Dong C ( 2008). TH17 cells in development: an updated view of their molecular identity and genetic programming. Nat Rev Immunol.

[4] Curotto de Lafaille MA, Lafaille JJ (2009). Natural and adaptive foxp3+ regulatory T cells: more of the same or a division of labor?. Immunity.

[5] Szabo SJ, Sullivan BM, Stemmann C ( 2002). Distinct effects of T-bet in TH1 lineage commitment and IFN-gamma production in CD4 and CD8 T cells. Science.

[6] Lieberman LA, Banica M, Reiner SL (2004). STAT1 plays a critical role in the regulation of antimicrobial effector mechanisms, but not in the development of Th1-type responses during toxoplasmosis. J Immunol.

[7] Arai KI, Lee F, Miyajima A ( 1990). Cytokines: coordinators of immune and inflammatory responses. Annu Rev Biochem.

[8] Zheng W, Flavell RA (1997). The transcription factor GATA-3 is necessary and sufficient for Th2 cytokine gene expression in CD4 T cells. Cell.

[9] Gordon S ( 2003). Alternative activation of macrophages. Nat Rev Immunol.

[10] Sato K, Mochizuki M, Saiki I ( 1994). Inhibition of tumor angiogenesis and metastasis by a saponin of Panax ginseng, ginsenoside-Rb2. Biol Pharm Bull.

[11] Kobayashi S, Miyamoto T, Kimura I (1995). Inhibitory effect of isoliquiritin, a compound in licorice root, on angiogenesis in vivo and tube formation in vitro. Biol Pharm Bull.

[12] Kim MS, Lee YM, Moon EJ ( 2000). Anti-angiogenic activity of torilin, a sesquiterpene compound isolated from Torilis japonica. Int J Cancer.

[13] Nagy JA, Dvorak AM, Dvorak HF (2003). VEGF-A(164/165) and PlGF: roles in angiogenesis and arteriogenesis. Trends Cardiovasc Med.

[14] Leelarungrayub N, Rattanapanone V, Chanarat N (2006). Quantitative evaluation of the antioxidant properties of garlic and shallot preparations. Nutrition.

[15] Yin M-c, Cheng W-s ( 1998). Antioxidant Activity of Several Allium Members. Journal of Agricultural and Food Chemistry.

[16] Ariga T, Seki T (2006). Antithrombotic and anticancer effects of garlic-derived sulfur compounds: a review. Biofactors.

[17] Jalal R, Bagheri SM, Moghimi A (2007). Hypoglycemic effect of aqueous shallot and garlic extracts in rats with fructose-induced insulin resistance. J Clin Biochem Nutr.

[18] Zhao M, Yang B, Wang J ( 2007). Immunomodulatory and anticancer activities of flavonoids extracted from litchi (Litchi chinensis Sonn) pericarp. Int Immunopharmacol.

[19] Nair MP, Kandaswami C, Mahajan S ( 2002). The flavonoid, quercetin, differentially regulates Th-1 (IFNgamma) and Th-2 (IL4) cytokine gene expression by normal peripheral blood mononuclear cells. Biochim Biophys Acta.

[20] Seyfi P, Mostafaie A, Mansouri K ( 2010). In vitro and in vivo anti-angiogenesis effect of shallot (Allium ascalonicum): a heat-stable and flavonoid-rich fraction of shallot extract potently inhibits angiogenesis. Toxicol In Vitro.

[21] Mohammadi-Motlagh HR, Mostafaie A, Mansouri K (2011). Anticancer and anti-inflammatory activities of shallot (Allium ascalonicum) extract. Arch Med Sci.

[22] Park EH, Joo MH, Kim SH (2003). Antiangiogenic activity of Gardenia jasminoides fruit. Phytother Res.

[23] He ZH, He MF, Ma SC ( 2009). Anti-angiogenic effects of rhubarb and its anthraquinone derivatives. J Ethnopharmacol.

[24] Mohammadi-Motlagh HR (2008). The study of anti-angiogenic effects of shallot (Allium hirtifolium) extract and isolation of effective fraction. Azarbayjan Univ Tarbiat Moallem.

[25] Mohammadi-Motlagh HR, Mansouri K, Shakiba Y (2009). Anti-angiogenic effect of aqueous extract of shallot (Allium ascalonicum) bulbs in rat aorta ring model. Yakhteh Med J.

[26] van de Loosdrecht AA, Beelen RH, Ossenkoppele GJ ( 1994). A tetrazolium-based colorimetric MTT assay to quantitate human monocyte mediated cytotoxicity against leukemic cells from cell lines and patients with acute myeloid leukemia. J Immunol Methods.

[27] Blecha F, Barry RA, Kelley KW ( 1982). Stress-induced alterations in delayed-type hypersensitivity to SRBC and contact sensitivity to DNFB in mice. Proc Soc Exp Biol Med.

[28] Mubarak AM, Kulatilleke CP ( 1990). Sulphur constituents of neem seed volatiles: A revision. Phytochemistry.

[29] Sengupta A, Ghosh S, Bhattacharjee S ( 2004). Allium vegetables in cancer prevention: an overview. Asian Pac J Cancer Prev.

[30] Yin MC, Tsao SM (1999). Inhibitory effect of seven Allium plants upon three Aspergillus species. Int J Food Microbiol.

[31] Ghodrati Azadi H, Ghaffari SM, Riazi GH ( 2008). Antiproliferative activity of chloroformic extract of Persian Shallot, Allium hirtifolium, on tumor cell lines. Cytotechnology.

[32] van Lier RA, ten Berge IJ, Gamadia LE (2003). Human CD8(+) T-cell differentiation in response to viruses. Nat Rev Immunol.

[33] Leighton T, Ginther C, Fluss L (1992). Molecular Characterization of Quercetin and Quercetin Glycosides in Allium Vegetables Phenolic Compounds in Food and Their Effects on Health II. American Chemical Society Inc.

[34] Fattorusso E, Iorizzi M, Lanzotti V ( 2002). Chemical composition of shallot (Allium ascalonicum Hort). J Agric Food Chem.

[35] Yu CS, Lai KC, Yang JS (2010). Quercetin inhibited murine leukemia WEHI-3 cells in vivo and promoted immune response. Phytother Res.

